# Impact of late gadolinium-enhanced cardiac MRI on arrhythmic and mortality outcomes in nonischemic dilated cardiomyopathy: updated systematic review and meta-analysis

**DOI:** 10.1038/s41598-023-41087-4

**Published:** 2023-08-23

**Authors:** Nonthikorn Theerasuwipakorn, Ronpichai Chokesuwattanaskul, Jeerath Phannajit, Apichai Marsukjai, Mananchaya Thapanasuta, Igor Klem, Pairoj Chattranukulchai

**Affiliations:** 1 Division of Cardiovascular Medicine, Department of Medicine, Faculty of Medicine, Chulalongkorn University, Cardiac Center, King Chulalongkorn Memorial Hospital, Bangkok, 10330 Thailand; 2 Division of Clinical Epidemiology, Department of Medicine, Faculty of Medicine, Chulalongkorn University, King Chulalongkorn Memorial Hospital, Bangkok, Thailand; 3 Division of Nephrology, Department of Medicine, Faculty of Medicine, Chulalongkorn University, King Chulalongkorn Memorial Hospital, Bangkok, Thailand; 4 Duke Cardiovascular Magnetic Resonance Center, Division of Cardiology, Duke University Medical Center, Durham, NC USA

**Keywords:** Outcomes research, Arrhythmias, Cardiomyopathies

## Abstract

Risk stratification based mainly on the impairment of left ventricular ejection fraction has limited performance in patients with nonischemic dilated cardiomyopathy (NIDCM). Evidence is rapidly growing for the impact of myocardial scar identified by late gadolinium enhancement (LGE) cardiac magnetic resonance imaging (CMR) on cardiovascular events. We aim to assess the prognostic value of LGE on long-term arrhythmic and mortality outcomes in patients with NIDCM. PubMed, Scopus, and Cochrane databases were searched from inception to January 21, 2022. Studies that included disease-specific subpopulations of NIDCM were excluded. Data were independently extracted and combined via random-effects meta-analysis using a generic inverse-variance strategy. Data from 60 studies comprising 15,217 patients were analyzed with a 3-year median follow-up. The presence of LGE was associated with major ventricular arrhythmic events (pooled OR: 3.99; 95% CI 3.08, 5.16), all-cause mortality (pooled OR: 2.14; 95% CI 1.81, 2.52), cardiovascular mortality (pooled OR 2.83; 95% CI 2.23, 3.60), and heart failure hospitalization (pooled OR: 2.53; 95% CI 1.78, 3.59). Real-world evidence suggests that the presence of LGE on CMR was a strong predictor of adverse long-term outcomes in patients with NIDCM. Scar assessment should be incorporated as a primary determinant in the patient selection criteria for primary prophylactic implantable cardioverter-defibrillator placement.

## Introduction

Cardiovascular complications particularly major ventricular arrhythmia and heart failure remain the leading causes of morbidity and mortality in patients with nonischemic dilated cardiomyopathy (NIDCM) despite advances in therapeutic strategies^1–3^. One of many efforts to reduce the risk of ventricular arrhythmia and death is the implantable cardioverter-defibrillator (ICD) insertion. For primary prevention, left ventricular ejection fraction (LVEF) ≤ 35% is the main selection criterion for ICD implantation in NIDCM patients^1^. However, there is growing evidence, that LVEF has significant limitations: (i) LVEF showed no or weak association with arrhythmic endpoints^4^; (ii) a recent clinical trial showed that a selection based on LVEF criteria failed to demonstrate mortality benefit^3^; (iii) less than one-third of ICD implanted patients with LVEF ≤ 35% had appropriate device therapy (ADT)^2^. Accordingly, LVEF ≤ 35% as an indication for primary ICD implantation has been downgraded from the class of recommendation I to IIa in the recent ESC Guidelines for the treatment of heart failure^5^ and the management of patients with ventricular arrhythmias and the prevention of sudden cardiac death^6^.

The pathophysiology of ventricular arrhythmia in NIDCM is frequently a reentry mechanism in the context of myocardial scar^1^. Late gadolinium enhancement (LGE) cardiovascular magnetic resonance imaging (CMR) is a noninvasive technique for the detection of scar in ischemic and non-ischemic cardiomyopathies. Over the past few years, a number of observational studies, as well as large, multicenter registries, have investigated the importance of LGE on CMR to predict adverse cardiovascular outcomes including cardiovascular mortality and ventricular arrhythmia^7–10^. These studies have shown that the presence of even a small area of LGE in patient with NIDCM has been associated with worse outcomes^8–10^. So far, no randomized clinical trial has demonstrated, however, that an intervention based on the information of CMR can reduce the risk of cardiovascular mortality. Guideline statements and Food and Drug Administration (FDA) approvals for medical devices have traditionally been based only on randomized clinical trials (RCT), however more recently the FDA is accepting real-world evidence (RWE) from registry data to aid in regulatory decision-making for medical device use^11^.

The aim of the present study was to perform a systematic review and meta-analysis to assess the predictive value of LGE on long-term outcomes in patients with NIDCM by utilizing the rapidly growing database and thus provide real-world evidence for consideration in regulatory decision-making.

## Methods

### Search strategy

This analysis was performed according to the Preferred Reporting Items for Systematic Reviews and Meta-analyses (PRISMA) statements and Meta-analysis of Observational Studies in Epidemiology (MOOSE)^12^. We (N.T., R.C.) conducted a systematic search of PubMed, Scopus, and Cochrane library databases from inception until 21 January 2022 for studies on the prognostic value of LGE in NIDCM. The references of included studies were reviewed for the completeness of the result. The search keywords were shown in Supplementary Data.

### Study eligibility

The inclusion criteria were: (i) prospective or retrospective cohort studies from patients diagnosed with NIDCM which were published in the peer-review, English-language journals; (ii) NIDCM definition fulfills the ESC guidelines for the diagnosis and treatment of acute and chronic heart failure diagnostic criteria based on LV dilatation and systolic dysfunction in the absence of known abnormal loading conditions or significant coronary artery disease^5^; (iii) studies with the available data on LGE presentation; (iv) mean follow-up time was longer than 6 months. Studies that included disease-specific subpopulations of NIDCM (e.g. hypertrophic or arrhythmogenic right ventricular cardiomyopathy, left ventricular non-compaction, infiltrative heart disease namely cardiac amyloidosis and sarcoidosis, acute myocarditis, drug- and toxin-induced cardiomyopathy, severe primary valvulopathy) were excluded. Editorials, reviews, conference abstracts, case reports, case series, systematic reviews, and meta-analyses were also excluded. Any disagreements concerning study choices were settled through collaborative conversation.

Two independent reviewers (N.T., R.C.) reviewed abstracts and full texts. The third reviewer (P.C.) will make the final decision when the consensus could not be determined. A study with the largest number of patients was selected for the analysis when two or more studies had an overlapping population.

### Data extraction and outcomes

Data extraction was performed by A.M. and M.T. The extracted data were first author, publication year, study site and country, study design, major inclusion, and exclusion criteria, LGE quantification and analysis methods, age, gender, comorbidities, New York Heart Association functional class, medications, and CMR parameters. Endpoints included in the meta-analysis were cardiovascular mortality (cardiovascular death, sudden cardiac death (SCD), and heart transplantation), major ventricular arrhythmic events (SCD, sustained ventricular tachycardia (VT), ventricular fibrillation (VF), and ADT), heart failure hospitalization, all-cause mortality (including heart transplantation), and major adverse cardiovascular events (MACE) by definition of the individual studies (when the definition was not provided, MACE was composite of all-cause mortality, heart transplantation, major ventricular arrhythmic events and heart failure hospitalization).

### Quality assessment

The modified Newcastle–Ottawa scale (NOS) for cohort studies was used to assess the quality of included studies based on eight domains categorized in three aspects: patient selection, comparability, and outcome. Two reviewers (A.M., M.T.) evaluated the study quality independently. Any disagreement was resolved by the consensus of the third reviewer (P.C.). Studies with a score of 6 or more were considered high-quality studies.

### Statistical analysis

All statistical analyses were performed using STATA version 16.1 (College Station, TX: StataCorp LLC.). The main analyses of each pre-specified outcome were performed using random-effect meta-analysis for binary outcomes using logarithmic odds-ratios (logOR) as effect size. The continuity correction of 0.5 was applied to studies with zero cells. DerSimonian and Laird’s generic inverse variance technique was used to calculate adjusted point estimates from each study, which assigned a weight to each study based on its variance^13^. In each analysis we reported the odds ratio and their 95% confidence intervals (95% CI) by exponentiating the logOR. The heterogeneity of the population was assessed by Cochran’s Q statistics and I^2^. The random-effect meta-regression was performed to examine the heterogeneity within the data. Funnel plots and the Egger test were utilized to assess the presence of publication bias^14^.

## Results

**Figure 1:**
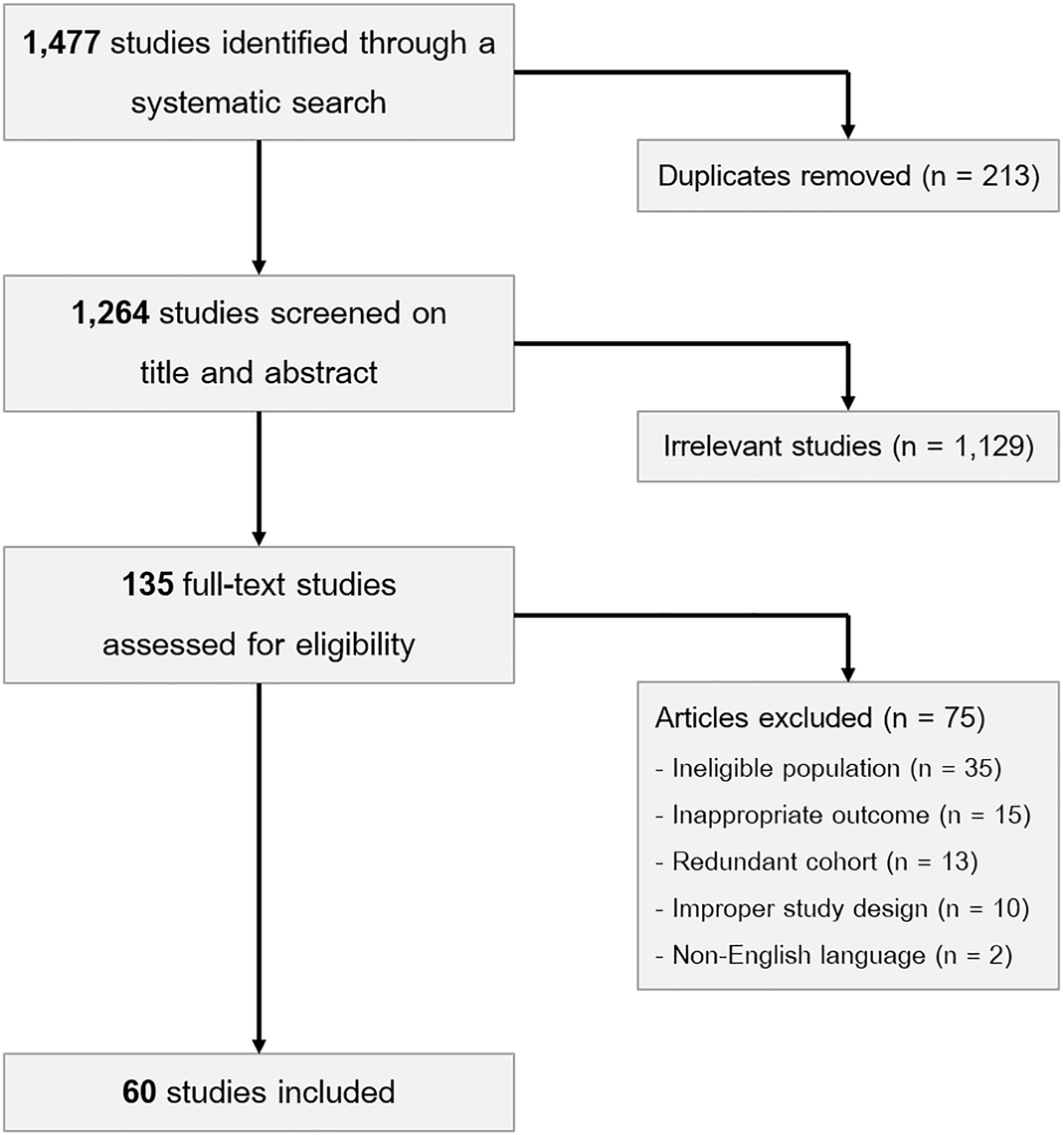
Flow diagram of studies searched in this meta-analysis.

A total of 1477 citations were acquired from a systematic search. Of these, 1342 citations were excluded by title and abstract screening, leaving 135 citations for full-text review. Seventy-five citations were excluded due to an ineligible population, redundant cohort, inappropriate outcome, non-English language, and improper study design. Finally, 60 studies were included in a systematic review (Fig. 1).

### Characteristics of included studies

Of 60 included studies, a total of 15,217 patients were enrolled with the number of participants in each study ranging from 31 to 1165 patients^4,9,10,15–71^. The median age was 54 years old (IQR: 50.0, 56.4). The proportion of males was 68.7%. The median follow-up time was 3.0 years (IQR: 1.8, 4.2). The median LVEF was 29.5% (IQR: 25.3, 35.8) and LGE was present in 7061 patients (46%, ranging from 25 to 82%) (Table 1).

**Table 1:** Baseline characteristics of included studies.

First author	Year	Study design	N	Inclusion criteria	Age (years)	LVEF (%)	LGE assessment	LGE present n (%)	Endpoint	Follow-up (year)
Wu^66^	2008	Prospective cohort	65	NIDCM, LVEF ≤ 35%, primary ICD prevention	55 ± 12	23.5 ± 10	Visualization*	27 (41.5%)	CV mortality, major VA event, HHF	1.4 + 17
Looi^42^	2010	Prospective cohort	103	NIDCM, LVEF < 50%, clinical HF	58 ± 13	32 ± 12	Visualization^†^	31 (30%)	All-cause mortality, major VA event, HHF	1.8 ± 0.9
Kono^37^	2010	Prospective cohort	32	NIDCM, LVEF < 40%	61.1 ± 11.5	21.3 ± 12	Intensity > 2 SD^†^	18 (56.3%)	All-cause mortality, CV mortality, major VA event, HHF	2.6 + 1.1
Cho^21^	2010	Prospective cohort	79	NIDCM, LVEF < 35%	56.4 ± 13.5	26.7 ± 8.4	Visualization^†^	42 (53.2%)	CV mortality, HHF	1.6 ± 0.8
Iles^32^	2011	Prospective cohort	61	NIDCM, advanced HF, primary ICD prevention	54 ± 13	26 ± 9	Intensity > 2 SD^†^	31 (61%)	Major VA event	1.6
Lehrke^38^	2011	Prospective cohort	184	NIDCM, LVEF < 50%	51.6 ± 1.1	31	Intensity > 2 SD*	72 (39.1%)	CV mortality, major VA event, HHF	1.8 ± 0.1
Gao^25^	2012	Prospective cohort	65	NIDCM, LVEF ≤ 35%	61 ± 11	25.5	Visualization, intensity, FWHM*	46 (70.8%)	Major VA event	1.7 ± 0.7
Gulati^28^	2013	Prospective cohort	472	NIDCM for at least 6 months, LVEF < 50%	51.1 ± 14.7	37.2 ± 13.1	FWHM*	142 (30.1%)	All-cause mortality, major VA event, HHF	5.3
Neilan^48^	2013	Prospective cohort	162	NIDCM, LVEF < 50%, primary ICD prevention	55 ± 14	28 ± 9	Visualization, intensity > 2 SD, FWHM*	81 (50%)	CV mortality, major VA event	2.2
Li^41^	2013	Retrospective cohort	293	NIDCM, LVEF < 50%	48.9 ± 15	33.5 ± 8.7	Visualization^†^	145 (49.5%)	All-cause mortality	3.2
Müller^46^	2013	Prospective cohort	185	Newly diagnosed NIDCM, clinical HF	51.2 ± 15.9	43.3 ± 16	Visualization^†^	94 (50.8%)	All-cause mortality, major VA event, HHF	1.75
Masci^44^	2014	Prospective cohort	228	NIDCM, no history of HF	50 ± 15	43 ± 10	Visualization^†^	61 (27%)	CV mortality, major VA event, HHF	1.9
Pöyhönen^54^	2014	Retrospective cohort	86	NIDCM, clinical HF	53	50	Visualization*	62 (70.9%)	MACE including CV mortality, major VA event	2.3
Rodríguez-Capitán^57^	2014	Retrospective cohort	64	NIDCM, LVEF < 50%	56.2 ± 13.4	29.1 ± 7.6	Visualization^†^	23 (35.9%)	CV mortality, major VA event, HHF	2.6
Machii^43^	2014	Retrospective cohort	72	NIDCM, LVEF < 45%, clinical HF	64 ± 14	24.8 ± 10.6	Intensity > 3 SD^†^	48 (67%)	CV mortality, major VA event, HHF	3 ± 1.7
Nabeta^47^	2014	Prospective cohort	76	Newly diagnosed NIDCM, LVEF < 45%	56 ± 13	30.2 ± 7.3	Intensity > 5 SD*	36 (47.4%)	MACE including major VA event, HHF	0.9 ± 0.3
Yamada^68^	2014	Prospective cohort	57	NIDCM, LVEF < 50%	55 ± 13	30 ± 11	Visualization, intensity > 2 SD*	25 (43.9%)	CV mortality, major VA event, HHF	5.9 ± 2.6
Perazzolo Marra^51^	2014	Prospective cohort	137	NIDCM, LVEF < 50%	49	36	Visualization, intensity > 2 SD*	76 (55.5%)	CV mortality, major VA event, HHF	3
Sadahiro^58^	2015	Retrospective cohort	76	NIDCM, LVEF < 45%, clinical HF	54 ± 14.9	21.9 ± 9.7	Visualization^†^	39 (51.3%)	All-cause mortality, CV mortality, HHF	2.22 ± 0.15
Tateishi^63^	2015	Prospective cohort	207	NIDCM, LVEF < 50%	50 ± 16	27 ± 11	Visualization^†^	105 (50.7%)	CV mortality, major VA event, HHF	3.6
Piers^53^	2015	Prospective cohort	87	NIDCM, LVEF < 50%, primary ICD prevention	56 ± 13	29 ± 12	Intensity > 35%*	55 (63%)	Major VA event	3.75
Venero^64^	2015	Retrospective cohort	31	Newly diagnosed NIDCM, LVEF ≤ 45%	46 ± 14	18 ± 8.8	Visualization^†^	18 (58%)	All-cause mortality, MACE including mortality, HHF	1
Chimura^19^	2015	Retrospective cohort	175	NIDCM, LVEF < 35%, clinical HF	60 + 15	29 + 5.4	Visualization^†^	122 (70%)	Major VA event	5.1 + 3.3
Gaztanaga^26^	2016	Retrospective cohort	105	NIDCM, LVEF ≤ 40%	51 ± 14	25.5 ± 9	Visualization, intensity > 2 SD*	71 (67.6%)	All-cause mortality, major VA event, HHF	2.2 ± 1.6
Shin^60^	2016	Retrospective cohort	365	NIDCM, LVEF < 50%, clinical HF	54.1 ± 14.5	26.5 ± 10.9	Visualization, FWHM*	261 (71.5%)	Major VA event	1.25
Puntmann^55^	2016	Prospective cohort	637	NIDCM	50	47	Visualization, FWHM*	171 (27%)	All-cause mortality	1.8
Hu^31^	2016	Prospective cohort	85	NIDCM, LVEF < 45%, clinical HF	56.5 ± 15.2	42 ± 13.6	Visualization, intensity*	35 (41.2%)	CV mortality, major VA event, HHF	7
Youn^70^	2017	Prospective cohort	117	NIDCM, LVEF ≤ 40%	51.9 ± 16.7	24.9 ± 8.1	Visualization, intensity > 5 SD*	82 (70.1%)	MACE including CV mortality, HHF	0.93
Halliday^10^	2017	Prospective cohort	399	NIDCM, LVEF ≥ 40%	49.9 ± 15.3	49.6 ± 4.9	Visualization, FWHM*	101 (25.3)	All-cause mortality, major VA event, MACE including CV mortality, HHF	4.6
Chimura^20^	2017	Retrospective cohort	179	NIDCM, LVEF < 50%	61 ± 15	33	Visualization^†^	100 (56%)	MACE including CV mortality, HHF	2.5
Arenja^15^	2017	Retrospective cohort	441	NIDCM, LVEF < 55%, clinical HF	53.5 ± 15.1	36.2 ± 12.9	Visualization^†^	185 (42%)	MACE including CV mortality, major VA event, HHF	4.2
Leyva^39^	2017	Retrospective cohort	252	NIDCM, clinical HF	66.6 ± 10	24.8 ± 12.4	Visualization^†^	68 (27.0%)	All-cause mortality, CV mortality, major VA event, HHF	3.8
Zhang^71^	2018	Prospective cohort	220	NIDCM, LVEF < 50%	49.5 ± 13.4	25.4 ± 10.4	Intensity > 2 SD^†^	101 (45.9%)	All-cause mortality, CV mortality, major VA event, HHF	5.1
Pi^52^	2018	Prospective cohort	172	NIDCM, LVEF < 40%	56.4 ± 14.3	23.7 ± 7.9	Visualization, intensity > 6 SD*	66 (38.4%)	All-cause mortality	3.9
Gutman^29^	2019	Prospective cohort	452	NIDCM, LVEF ≤ 35%, clinical HF	53.4	25.2	Visualization^†^	277 (61.3%)	All-cause mortality, CV mortality	3.2
Vita^65^	2019	Retrospective cohort	240	NIDCM, LVEF < 60%, clinical HF	49 ± 16	43 ± 15	Visualization, intensity > 4 SD*	81 (35%)	MACE including all-cause mortality, HHF	3.8 ± 1.6
Sree Raman^62^	2019	Prospective cohort	49	NIDCM, LVEF ≤ 45%, clinical HF	61	20	Visualization^†^	17 (34.7%)	CV mortality, major VA event, HHF	8.2
Halliday^30^	2019	Prospective cohort	874	NIDCM, LVEF < 50%	53.4 ± 14.7	36.4 ± 12.7	Visualization, FWHM*	300 (34.3%)	All-cause mortality, major VA event	4.9
Yi^69^	2020	Retrospective cohort	378	NIDCM, LVEF < 50%, clinical HF	55 ± 15	24.1 ± 8.9	Visualization, FWHM*	258 (68.3%)	MACE including all-cause mortality, major VA event, HHF	3.4 ± 3
Cojan-Minzat^23^	2020	Prospective cohort	178	Newly diagnosed NIDCM, LVEF ≤ 45%	48 ± 14.4	35 ± 9.3	Intensity > 5 SD*	64 (36.0%)	MACE including major VA event, HHF	1.4
Behera^17^	2020	Retrospective cohort	112	NIDCM, LVEF < 50%	40	21	Intensity > 2 SD*	44 (39%)	All-cause mortality, CV mortality, major VA event, HHF	2 ± 0.9
Barison^16^	2020	Retrospective cohort	183	NIDCM, primary ICD prevention	66	27	Visualization, intensity > 6 SD*	116 (63%)	Major VA event	2.5
Elming^9^	2020	Prospective cohort	236	NIDCM, LVEF ≤ 35%, NT-proBNP > 200 pg/mL	61	33	Visualization, FWHM*	113 (47.9%)	All-cause mortality, CV mortality, major VA event	5.3
Cittar^22^	2021	Retrospective cohort	273	NIDCM, LVEF < 50%	51	34	Visualization^†^	140 (52%)	MACE including CV mortality, major VA event	3.25
Ota^50^	2021	Retrospective cohort	101	NIDCM, LVEF < 50%, clinical HF	61.2 ± 12.3	32.3 ± 9.3	Visualization, intensity > 5 SD*	53 (52.5%)	MACE including CV mortality, major VA event, HHF	5.4
Infante^33^	2021	Retrospective cohort	86	NIDCM, LVEF ≤ 50%	44.9 ± 16.1	36.9 ± 12.2	Visualization^†^	55 (64%)	CV mortality, major VA event, HHF	4.9 ± 3.2
Kolluru^36^	2021	Prospective cohort	61	NIDCM, LVEF ≤ 40%, clinical HF	54 ± 13	33	Visualization, intensity > 2.5 SD*	21 (34.4%)	MACE including CV mortality, major VA event	2 ± 0.3
Kim^34^	2021	Retrospective cohort	78	NIDCM, LVEF < 35%, clinical HF	54.9 ± 13.6	25.4	Intensity > 5 SD*	63 (80.8%)	MACE including CV mortality, major VA event, HHF	3
Chen^18^	2021	Retrospective cohort	157	NIDCM, LVEF ≤ 50%	52.3 ± 16.1	27 ± 10.7	Visualization, intensity > 5 SD*	121 (77.1%)	All-cause mortality, major VA event	1.1
Klem^35^	2021	Prospective cohort	1020	NIDCM, LVEF < 50%	54	33	Visualization, intensity > 2 SD*	461 (45.2%)	All-cause mortality, CV mortality, major VA event	5.2
Xu^67^	2021	Prospective cohort	412	NIDCM	48 ± 14.4	23.7 ± 9.8	Visualization, FWHM*	201 (48.8%)	All-cause mortality	2.3
Ochs^49^	2021	Retrospective cohort	350	NIDCM, LVEF ≤ 45%	52.2 ± 15.2	36.4 ± 13.7	Visualization^†^	134 (38.3%)	MACE including CV mortality, major VA event	4.2
Raafs^56^	2021	Prospective cohort	209	NIDCM, LVEF < 50%	54 ± 13	34 ± 12	Visualization, FWHM*	65 (31%)	MACE including all-cause mortality, major VA event, HHF	6.3
Fu^24^	2021	Retrospective cohort	126	NIDCM, LVEF < 40%	49.9 ± 15.8	22.3 ± 8.1	Intensity > 5 SD*	66 (52.4%)	MACE including CV mortality, HHF	2.5
Mikami^45^	2021	Prospective cohort	645	NIDCM, LVEF ≤ 50%	56 ± 14	37 ± 11	Visualization^†^	306 (47%)	MACE including all-cause mortality, HHF	2.9
Shams^59^	2021	Retrospective cohort	75	NIDCM, LVEF < 45%	38.7 ± 13	29.3 ± 12	Visualization^†^	28 (37.3%)	MACE including all-cause mortality, major VA event, HHF	3.3 ± 2.3
Shu^61^	2021	Retrospective cohort	129	NIDCM, LVEF < 35%	47	15.33	Intensity > 6 SD*	97 (82.2%)	MACE including all-cause mortality, major VA event	1.4
Guaricci^27^	2021	Prospective cohort	1000	NIDCM, LVEF < 50%	56.7 ± 14.2	33.4 ± 10.9	Visualization^†^	457 (46%)	All-cause mortality, major VA event	2.6
Di Marco^4^	2021	Retrospective cohort	1165	NIDCM, LVEF < 50%, nonischemic non-dilated cardiomyopathy	58	39	Visualization^†^	486 (41.7%)	Major VA event	3
Li^40^	2022	Retrospective cohort	659	NIDCM, LVEF < 45%	45 ± 15	29.6 ± 9.3	FWHM*	355 (55.9%)	CV mortality	5.4 ± 1.8

*Studies reported the extent of myocardial scar (late gadolinium enhancement quantification).

^†^Studies reported the presence or absence of late gadolinium enhancement.

*LGE* late gadolinium enhancement, *LVEF* left ventricular ejection fraction, *NIDCM* nonischemic dilated cardiomyopathy, *ICD* implantable cardioverter-defibrillator, *CV* cardiovascular, *HHF* hospitalized heart failure, *MACE* major adverse cardiovascular events, *VA* ventricular arrhythmia, *FWHM* full width at half maximum.

### LGE and major ventricular arrhythmic events

**Figure 2:**
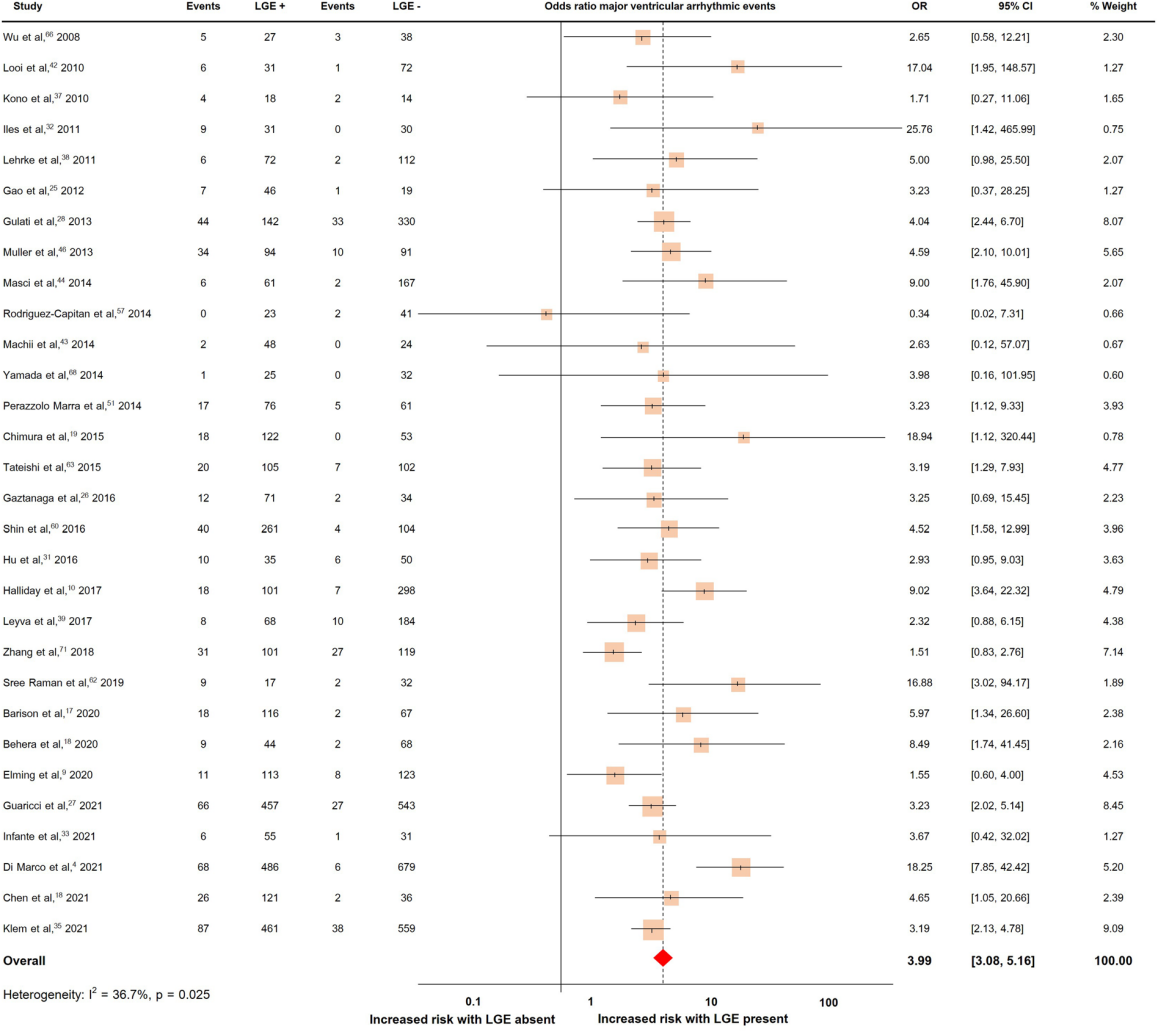
Forrest plot illustrating the risk of major ventricular arrhythmic events in individual studies.

Thirty studies with a total of 7541 patients reported major ventricular arrhythmic events, which occurred in 810 patients (10.7%)^4,9,16–19,25–28,31–33,35,37–39,42–44,46,51,57,60,62,63,66,68,71^. The pooled OR and rates of major ventricular arrhythmic events were shown in Fig. 2. The presence of LGE predicted major ventricular arrhythmic events with a pooled OR of 3.99 (95% CI 3.08, 5.16). The heterogeneity (I^2^) was 36.7% (p = 0.025).

### LGE and all-cause mortality

**Figure 3:**
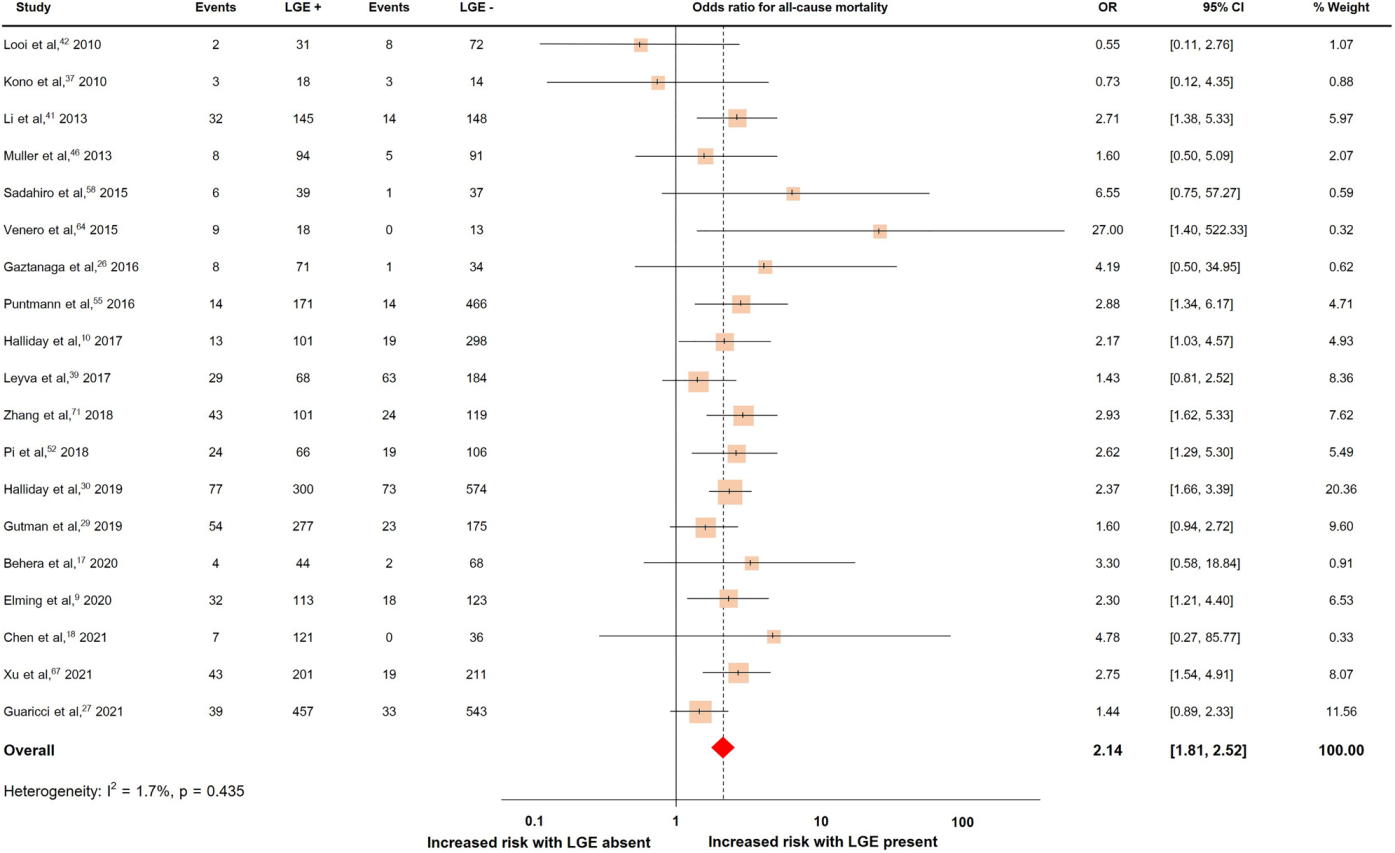
Forrest plot illustrating the risk of all-cause mortality in individual studies.

Nineteen studies with a total of 5748 patients reported all-cause mortality, which occurred in 786 patients (13.7%)^9,10,17,18,26,27,29,30,37,39,41,42,46,52,55,58,64,67,71^. The pooled OR and rates of all-cause mortality were shown in Fig. 3. The presence of LGE predicted all-cause mortality with a pooled OR of 2.14 (95% CI 1.81, 2.52). The heterogeneity (I^2^) was 1.7% (p = 0.435).

### LGE and cardiovascular mortality

**Figure 4:**
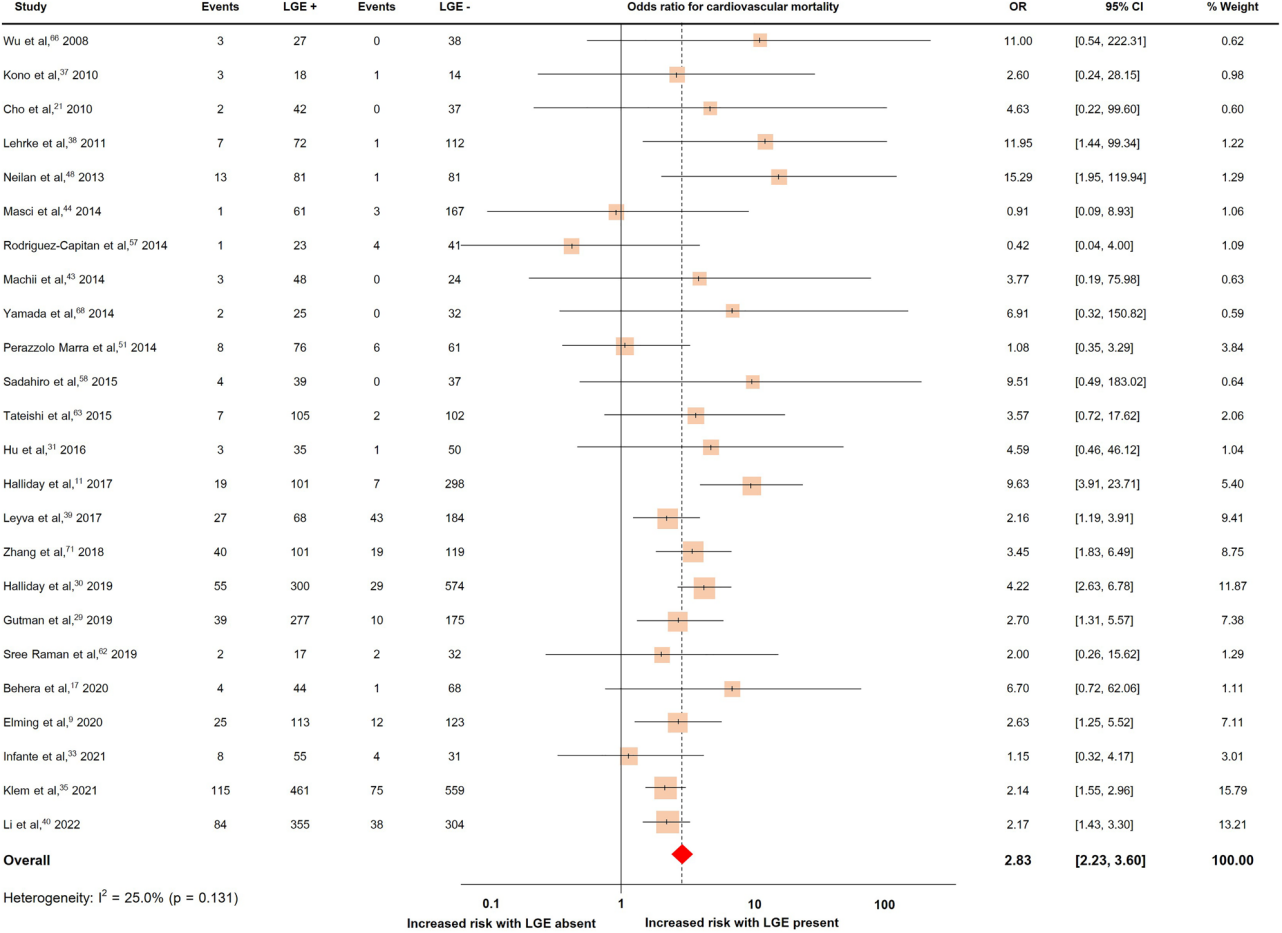
Forrest plot illustrating the risk of cardiovascular mortality in individual studies.

Twenty-four studies with a total of 5807 patients reported cardiovascular mortality, which occurred in 734 patients (12.6%)^9,10,17,21,29–31,33,35,37–40,43,44,48,51,57,58,62,63,66,68,71^. The pooled OR and rates of cardiovascular mortality were shown in Fig. 4. The presence of LGE predicted cardiovascular mortality with a pooled OR of 2.83 (95%CI 2.23, 3.60). The heterogeneity (I^2^) was 25.0% (p = 0.131).

### LGE and heart failure hospitalization

**Figure 5:**
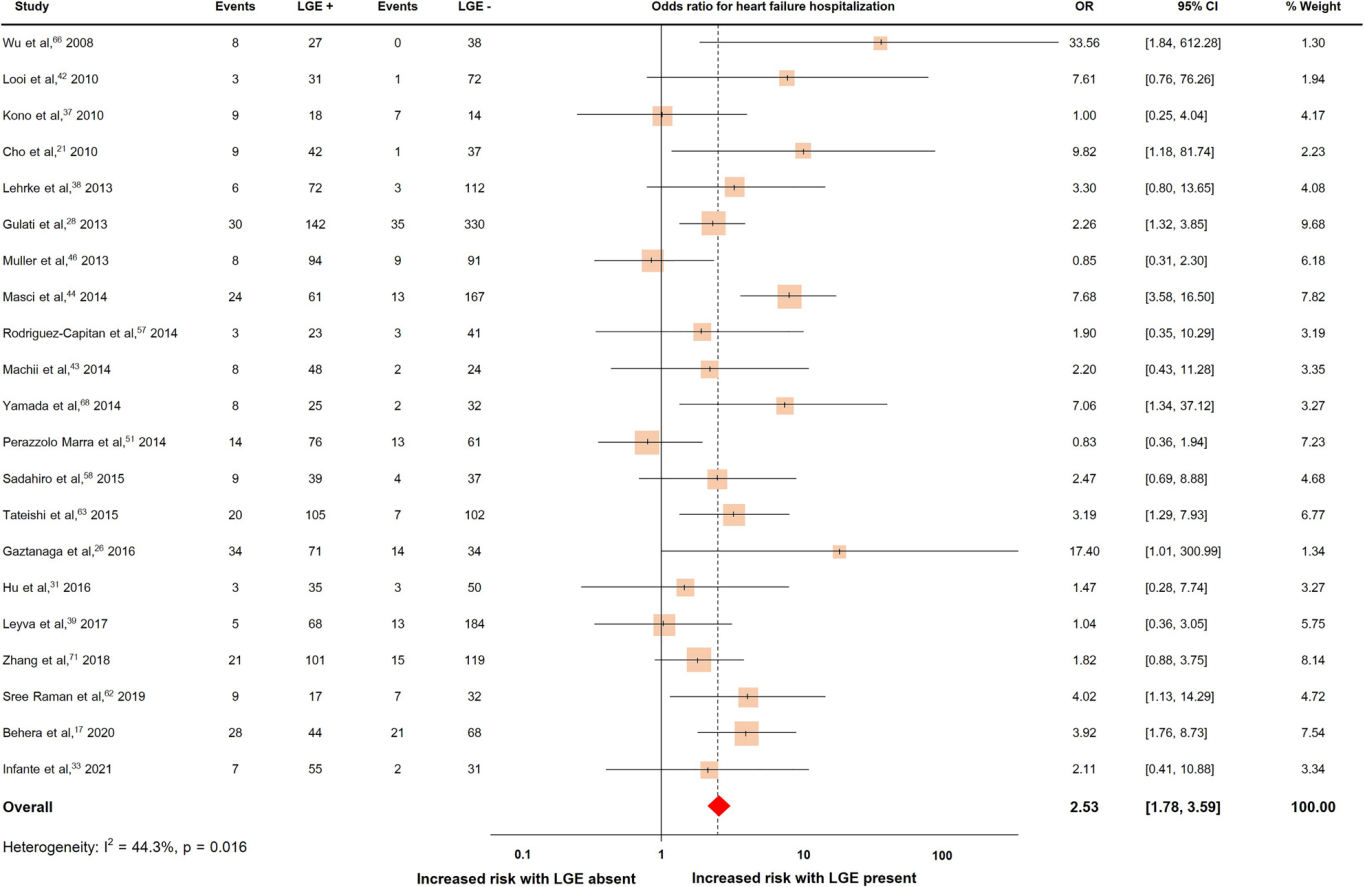
Forrest plot illustrating the risk of heart failure hospitalization in individual studies.

Twenty-one studies with a total of 2870 patients reported heart failure hospitalization, which occurred in 407 patients (14.2%)^17,21,26,28,31,33,37–39,42–44,46,57,58,62,63,66,68,71^. The pooled OR and rates of heart failure hospitalization were shown in Fig. 5. The presence of LGE predicted heart failure hospitalization with a pooled OR of 2.53 (95% CI 1.78, 3.59). The heterogeneity (I^2^) was 44.3% (p = 0.016).

### LGE and major adverse cardiac events

**Figure 6:**
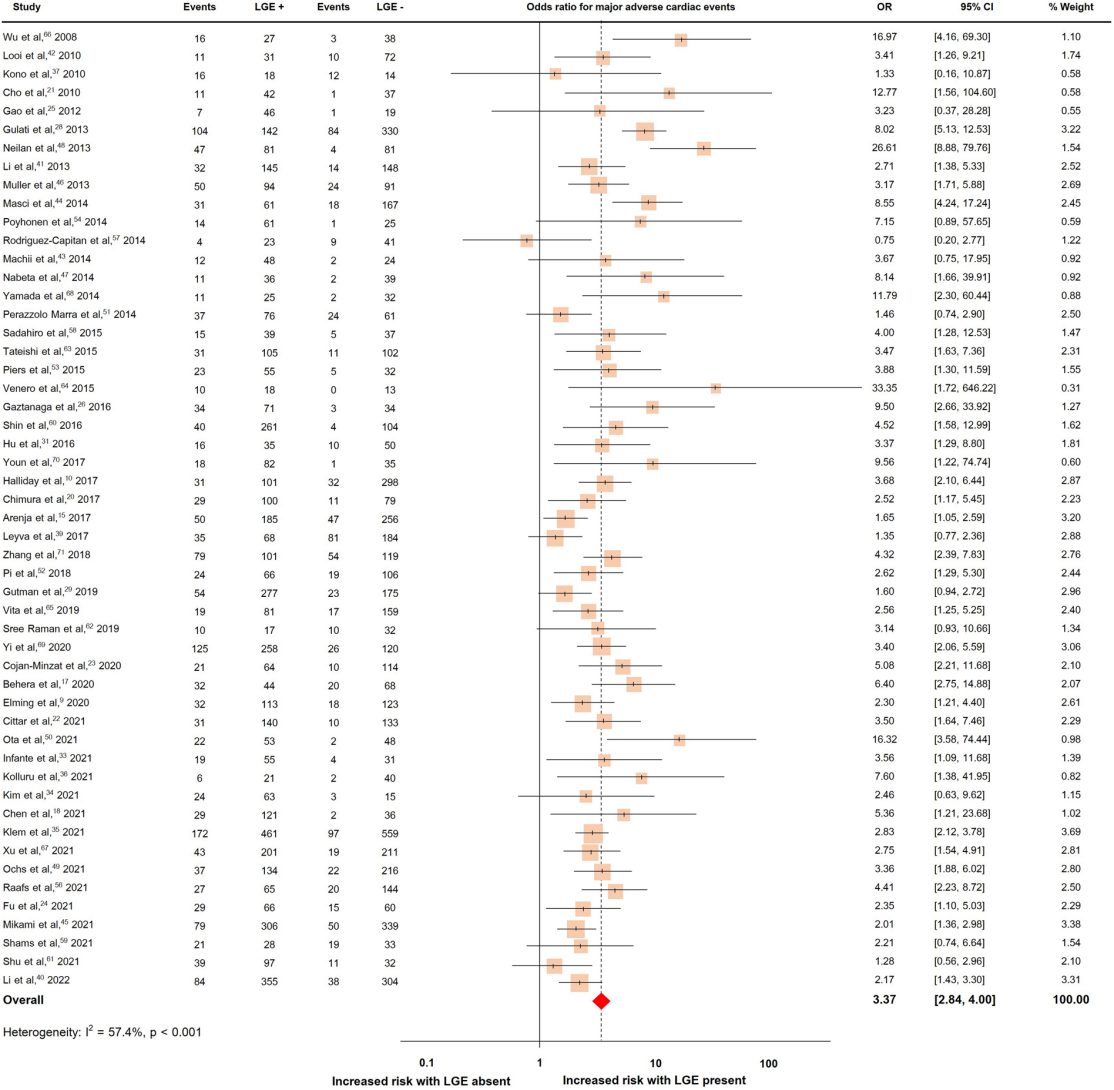
Forrest plot illustrating the risk of major adverse cardiac events in individual studies.

Fifty-two studies with a total of 10,923 patients reported MACE, which occurred in 2736 patients (25.1%)^9,10,15,17,18,20–26,28,29,31,33–37,39–54,56–71^. The pooled OR and rates of MACE were shown in Fig. 6. The presence of LGE predicted MACE with a pooled OR of 3.37 (95% CI 2.84, 4.00). The heterogeneity (I^2^) was 57.4% (p < 0.001).

### Meta-regression

The meta-regression results revealed no significant association between ORs in the main studies and LVEF or LGE extent for all adverse outcomes. However, a statistically significant negative correlation was observed between the effect sizes of all-cause mortality and age (log odds − 0.04, 95% CI  − 0.07, − 0.01; p = 0.01) (Supplementary Table 1).

### Quality assessment

All included studies had a NOS score of 6 or more and were considered high-quality studies. Forty-five studies (75%) had a follow-up time of more than 2 years (Supplementary Table 2).

### Evaluation of publication bias

The funnel plots of all outcomes appeared asymmetrical (Figs. A–E in the Supplement). The Egger test showed a presence of publication bias (p 0.039).

## Discussion

From the current meta-analysis, we found that the presence of LGE on CMR predicts all major clinical outcomes in patients with NIDCM. During the median follow-up time of 3 years, the pooled ORs were 3.99 (95% CI 3.08, 5.16) for major ventricular arrhythmic events, 2.14 (95% CI 1.81, 2.52) for all-cause mortality, 2.83 (95% CI 2.23, 3.60) for cardiovascular mortality, 2.53 (95% CI 1.78, 3.59) for heart failure hospitalization, and 3.37 (95% CI 2.84, 4.00) for MACE. The present meta-analysis reflects the rapidly growing evidence of LGE for risk stratification in NIDCM including 60 studies with over 15,000 patients, some were very recent large, multi-center registries with over 1000 subjects^35^. The average LGE prevalence was 46%, ranging from 25 to 82%. The LGE quantification techniques used were quite heterogenous between studies, some using the standard deviation of a signal of normal myocardium (e.g. > 2, 2.5, 3, 5 and 6SD) intensity thresholding method, others using the full width at half maximum method and some used visual scoring of LGE extent. Also, the metric system of LGE extent was various; for example, 26 studies used the percentage of LGE compared to the total LV mass (reported range from 2.1 to 17.2%) and 8 studies used absolute extent as a gram of LGE (reported range from 2.9 to 34.5 g). Furthermore, 26 studies did not quantify the extent of LGE (Table 1). Despite the existence of a quantitative relationship between the LGE extent and the increase in arrhythmic risk^35^, the cutoff threshold for LGE extent (expressed as a percentage of LV mass) and its associated risk has not been determined yet. This is partly due to the use of different quantification methods in the literature. Additionally, a direct comparison to demonstrate the prognostic value between evaluations based on LGE extent and those based on the presence or absence of LGE has not been conducted. Nonetheless, we consider evaluating the presence or absence of LGE to be a practically reasonable and validated risk marker at present. The mere presence of LGE has been associated with a 2.8-fold higher cardiovascular mortality risk and nearly fourfold higher risk for arrhythmic events. Further studies are warranted to refine the optimal LGE quantification technique and determine the LGE extent for improved risk stratification.

To date, the guideline recommendation for primary ICD insertion in patients with NIDCM is depending mainly on LVEF of 35% or less^1,5^. The role of LGE on CMR has been acknowledged primarily as an additional risk factor that should be considered in conjunction with impaired LVEF when contemplating ICD implantation in the latest guideline (class IIa)^6^. However, many publications showed that LVEF might not be an appropriate prognosticator^3,4,72^. Halliday et al.^10^ conducted a prospective cohort study specifically focusing on patients with NIDCM and mild to moderate LV systolic dysfunction, including only patients with LVEF ≥ 40%. The incidence of the primary composite endpoint, which comprised SCD and aborted SCD (defined as major ventricular arrhythmic events in our study), was 6%. Notably, the incidence was significantly higher at 17.8% in patients with LGE, compared to 2.3% in patients without LGE. On the contrary, LGE on CMR, as a representative of myocardial fibrosis, has emerged as an important risk marker whether based on arrhythmic pathophysiology or evidence from recent studies^7,8,73^. Furthermore, LGE is a highly consistent risk marker because once it is present on CMR, it does not regress in size or resolve over time^74^.

The previous systemic review and meta-analysis by Di Marco et al. in 2017^75^ and by Becker et al. in 2018^7^ nicely reported the valuable prognostic tool of LGE in NIDCM patients. Given the exponential growth of studies with a large sample size published in the past few years, the present meta-analysis, which utilized the rapidly growing database available in 2022, strengthened the role of LGE in identifying NIDCM patients at risk of future adverse events. It is important to highlight the fact that we included 15,217 patients from 60 studies compared with 4554 patients from 34 studies as reported by Becker et al. A substantial number of patients provided an adequate number of individuals per each analytic outcome. Hence, we could assess all major clinical endpoints including all-cause mortality that was not reported in the recent meta-analysis^7^. By comparing the results, we found a very similar OR for heart failure hospitalizations compared with the study by Becker et al. (2.53 vs 2.66). In addition, despite including more than the double of patients than the study by Di Marco et al.^75^, the pooled OR for major ventricular arrhythmic events was very similar (3.99 vs 4.3). The new larger analysis has largely confirmed the findings of smaller prior ones with consistent results. These emphasize the strength of the association between LGE and specific cardiovascular events.

The meta-regression results revealed no significant association between ORs in the main studies and LVEF or LGE extent for all adverse outcomes. However, a statistically significant negative correlation was observed between the effect sizes of all-cause mortality and age. This indicates that the presence of LGE is more strongly linked to all-cause mortality in a younger population. Our hypothesis is that in an older population, the likelihood of death from non-cardiovascular causes is higher, which diminishes the impact of LGE. This hypothesis is supported by the insignificant meta-regression results of age in other cardiovascular outcomes.

For many years, it has been widely accepted that well-designed RCTs are warranted to provide the best evidence for refining the indication for prophylactic ICD in patients with NIDCM. However, the FDA has already accepted the RWE from registry data to aid in regulatory decision-making for medical device implantation^11^. While we are still waiting for the results of using the presence of LGE as guidance for ICD implantation from an ongoing multicenter RCT that has just started enrolling subjects^76^, based on the robust findings derived from the present meta-analysis, which encompasses a substantial number of patients, we believe that these results can enhance the importance of LGE assessment as a primary determinant, transcending its current contributory role.

## Limitation

This meta-analysis has some limitations. First, although we performed an extensive systematic search via several large databases, the results are still subjected to publication bias as demonstrated by asymmetrical funnel plots and the Egger test result. Second, there was population heterogeneity in the analysis for major ventricular arrhythmic events, heart failure hospitalization, and MACE. Even though we included only studies focused on NIDCM, the inclusion and exclusion criteria, magnetic field strength, contrast type and dosage, and also pulse sequence used for LGE analysis in the individual studies are varied. Thus, we used the random-effect model in our meta-analysis for this reason. Third, most of the included studies were retrospective and had a small number of participants e.g. 38 studies (61%) had participants of less than 200. Nevertheless, all studies had NOS scores of 6 or more, which are considered high-quality studies and could strengthen the results. Fourth, in some patients with NIDCM, the LGE extent may increase over time, and progressive disease is associated with a particularly high risk^74^. Therefore, a quantitative assessment of LGE on CMR may be necessary to evaluate the progressive condition. Lastly, LGE on CMR only detected focal and dense but not diffuse and interstitial fibrosis. Newer techniques e.g. T1 mapping, which showed promising result in detecting diffuse fibrosis may provide additional prognostic information in patients with NIDCM.

## Conclusion

Real-world evidence suggests that the presence of LGE on CMR was a strong predictor of adverse outcomes including mortalities, major ventricular arrhythmic events, heart failure hospitalization, and MACE in patients with NIDCM. Scar assessment should be incorporated as a primary determinant in the patient selection criteria for primary prophylactic ICD placement.

### Supplementary Information

Supplementary Information.

## Data Availability

All data generated or analyzed during this study are included in this published article and in its supplementary information file. The processed data are available from the corresponding author upon request.
