# Supplementary material for: Impact of late gadolinium-enhanced cardiac MRI on arrhythmic and mortality outcomes in nonischemic dilated cardiomyopathy: updated systematic review and meta-analysis
Source: Sci Rep. 2023 Aug 23;13:13775. doi: 10.1038/s41598-023-41087-4 (PMC10447440; doi:10.1038/s41598-023-41087-4)
Supplement: Supplementary file 1 — Supplementary Information. [file 41598_2023_41087_MOESM1_ESM.docx]

**Supplementary data**: The searching keywords for systematic reviews

[(DCM OR NIDCM OR nonischemic dilated cardiomyopathy OR nonischemic cardiomyopathy OR idiopathic dilated cardiomyopathy OR dilated cardiomyopathy)] AND [(CMR OR cardiac magnetic resonance imaging)] AND [(LGE OR late gadolinium enhancement OR delayed gadolinium enhancement OR contrast enhancement OR gadolinium enhancement OR delayed enhancement OR scar OR fibrosis)] AND [(Prognosis OR risk assessment OR risk stratification OR predictive value OR outcome OR MACE OR major adverse cardiovascular event OR death OR SCD OR sudden cardiac death OR heart transplantation OR VA OR “ventricular arrhythmia” OR VF OR “ventricular fibrillation” OR VT OR “ventricular tachycardia” OR “ICD therapy” OR heart failure OR hospitalization)]

**Supplementary Table 1: Meta-regression**

**
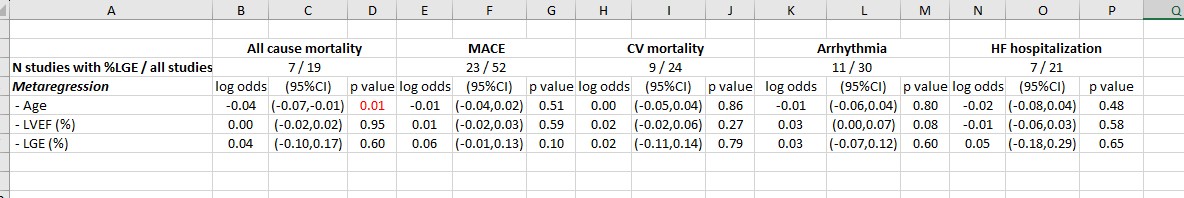
**

**Supplementary Table 2:** The modified Newcastle-Ottawa scale (NOS) score for quality assessment of each study


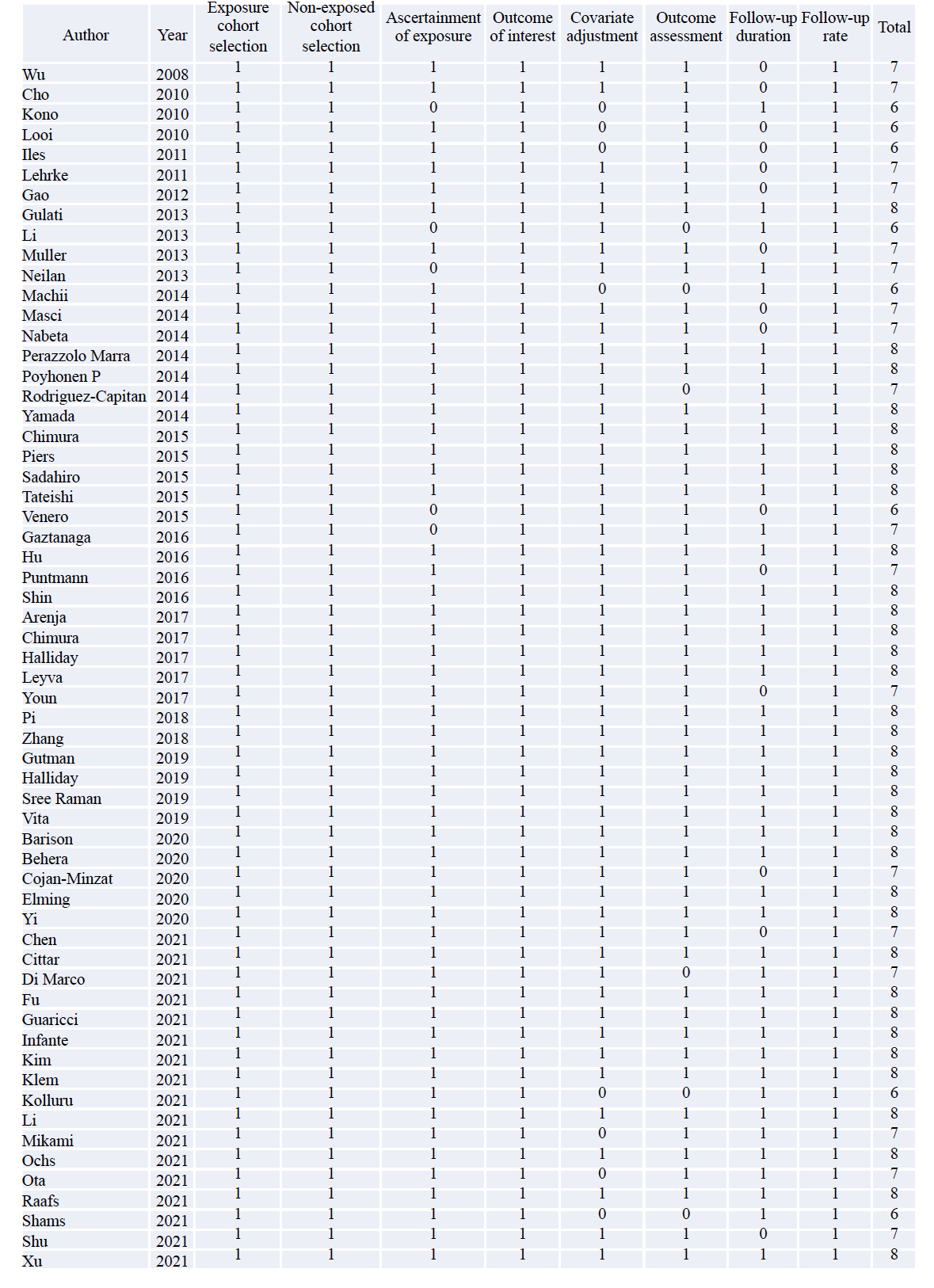


**
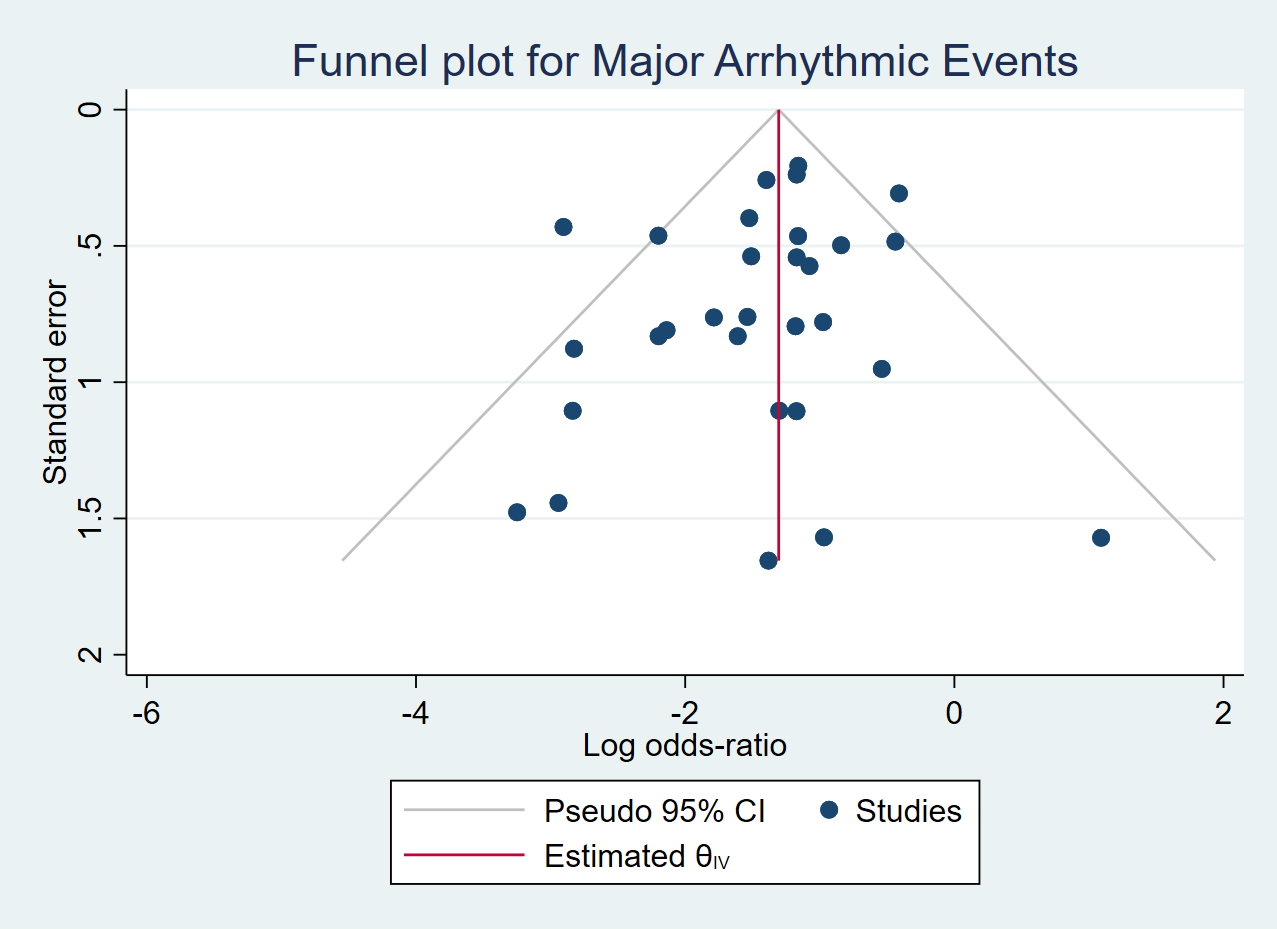
**

**Supplementary Figure A:** Evaluation of publication bias for major arrhythmic events


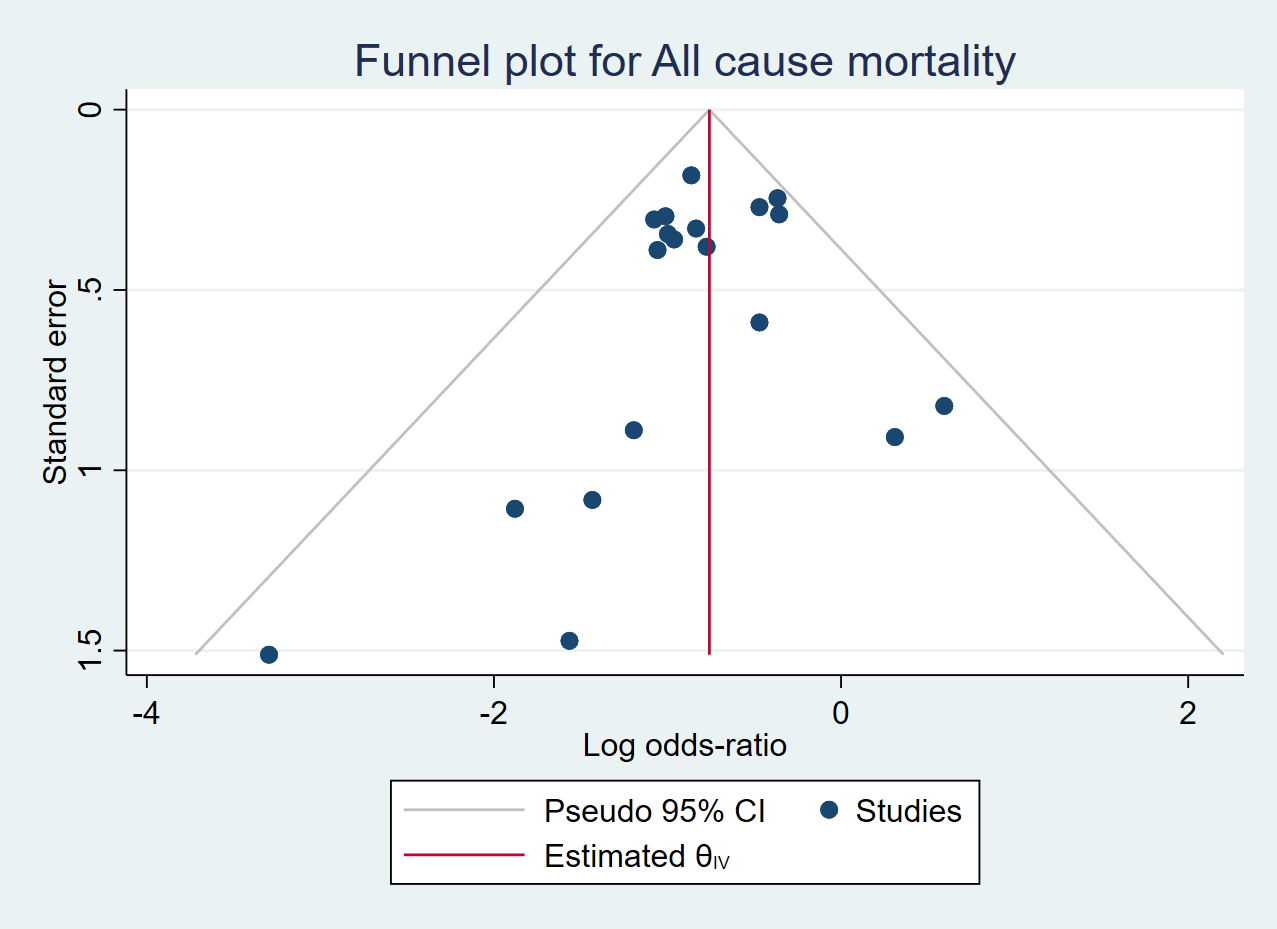


**Supplementary Figure B:** Evaluation of publication bias for all cause mortality


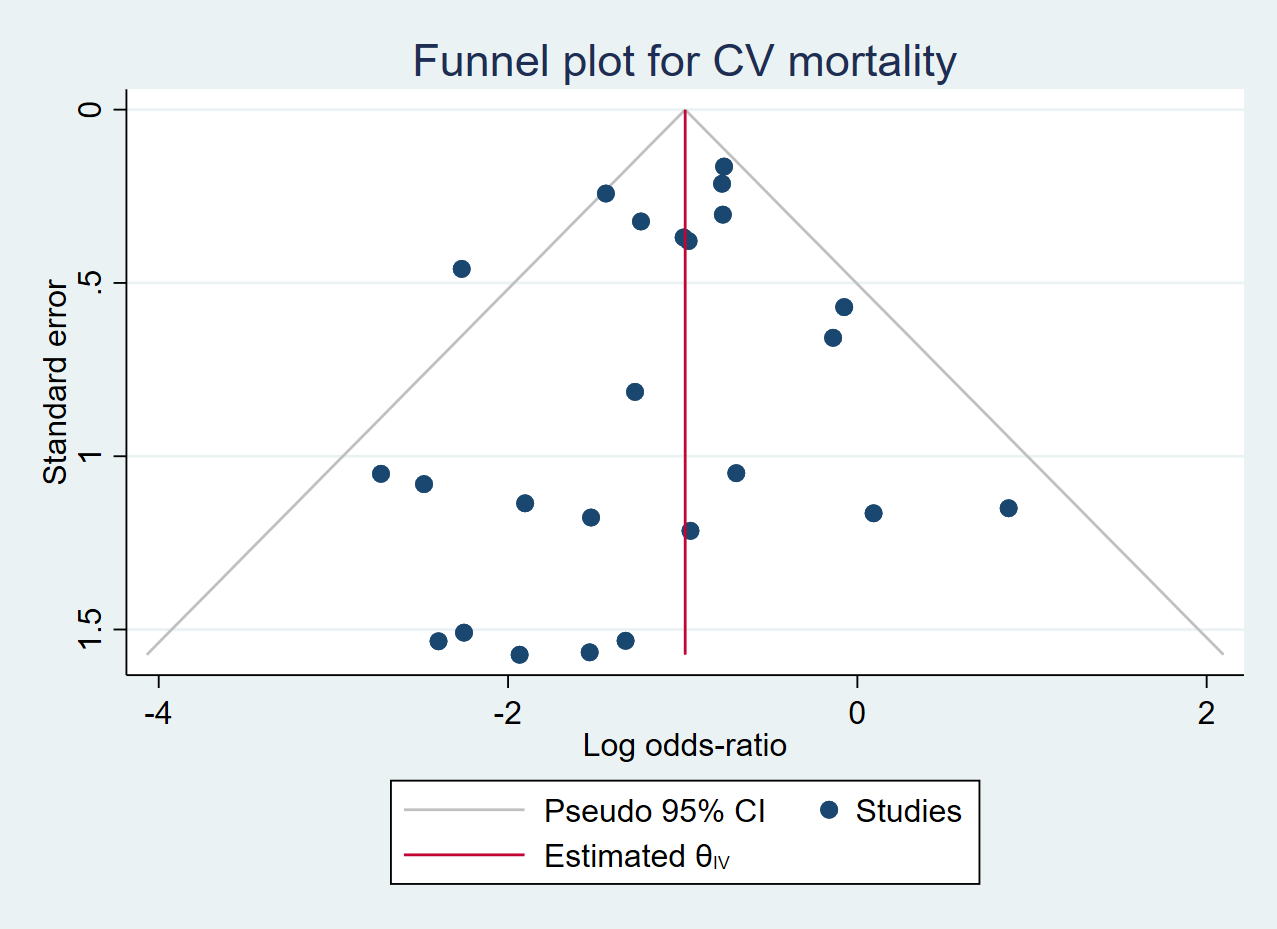


**Supplementary Figure C:** Evaluation of publication bias for cardiovascular mortality


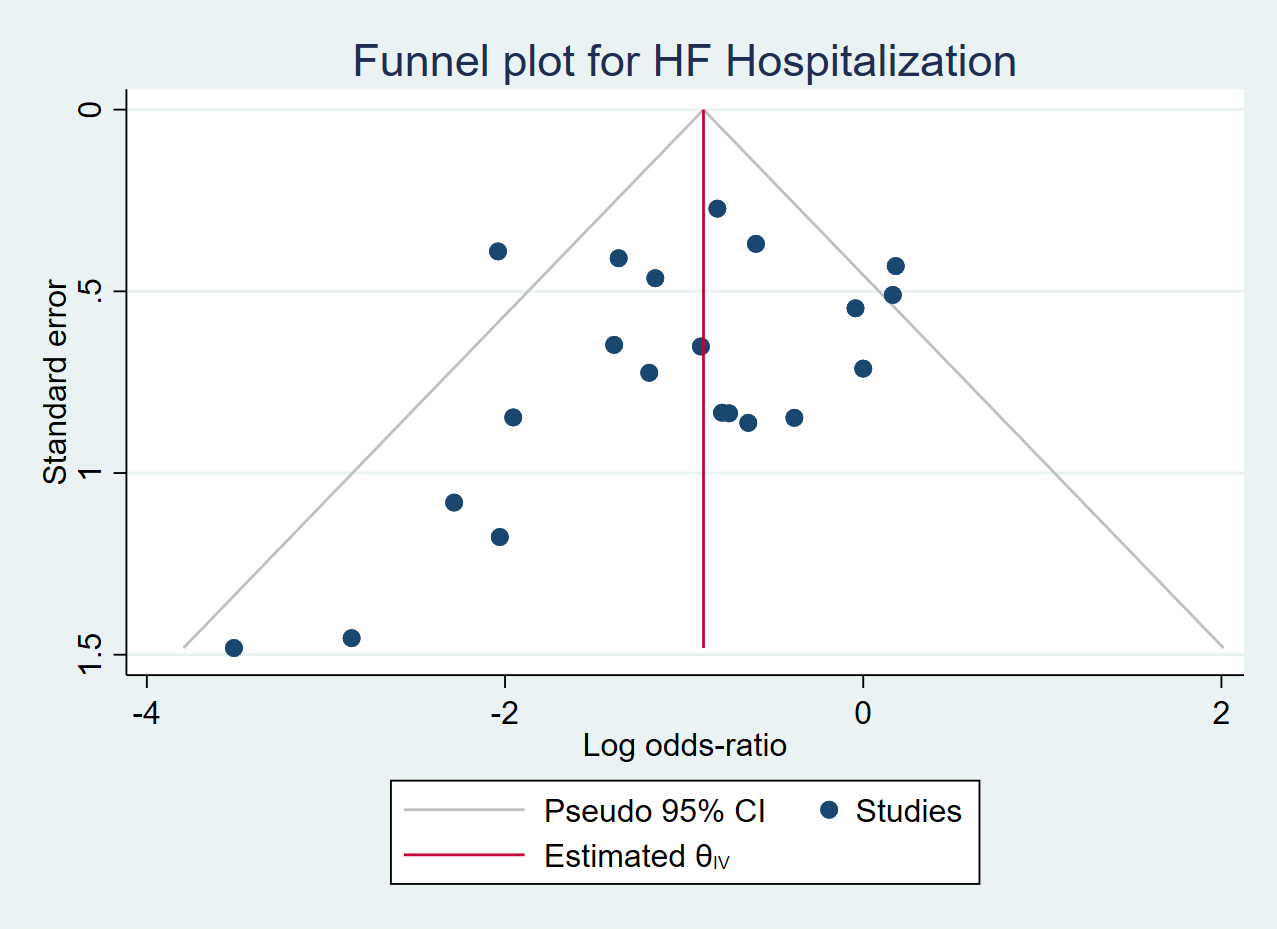


**Supplementary Figure D:** Evaluation of publication bias for heart failure hospitalization

**
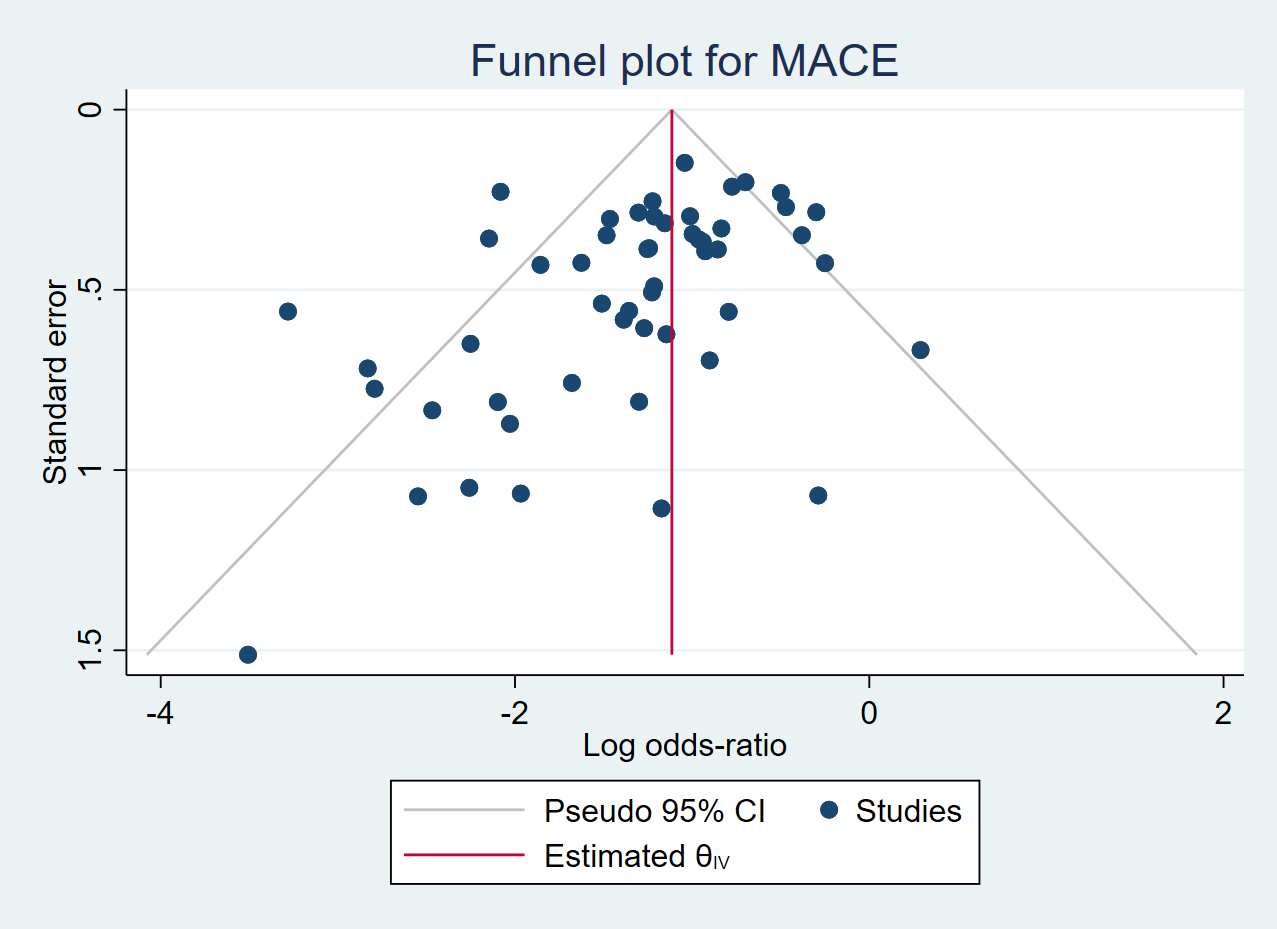
**

**Supplementary Figure E:** Evaluation of publication bias for major adverse cardiovascular events
